# Distinct Characteristics of Circulating Vascular Endothelial Growth Factor-A and C Levels in Human Subjects

**DOI:** 10.1371/journal.pone.0029351

**Published:** 2011-12-20

**Authors:** Hiromichi Wada, Shuichi Ura, Shuji Kitaoka, Noriko Satoh-Asahara, Takahiro Horie, Koh Ono, Tomohide Takaya, Rieko Takanabe-Mori, Masaharu Akao, Mitsuru Abe, Tatsuya Morimoto, Toshinori Murayama, Masayuki Yokode, Masatoshi Fujita, Akira Shimatsu, Koji Hasegawa

**Affiliations:** 1 Division of Translational Research, National Hospital Organization Kyoto Medical Center, Kyoto, Japan; 2 Health Evaluation Center, National Hospital Organization Kyoto Medical Center, Kyoto, Japan; 3 Division of Diabetic Research, National Hospital Organization Kyoto Medical Center, Kyoto, Japan; 4 Department of Clinical Innovative Medicine, Translational Research Center, Kyoto University Hospital, Kyoto, Japan; 5 Department of Cardiovascular Medicine, Graduate School of Medicine, Kyoto University, Kyoto, Japan; 6 Department of Cardiology, National Hospital Organization Kyoto Medical Center, Kyoto, Japan; 7 Division of Molecular Medicine, School of Pharmaceutical Sciences, University of Shizuoka, Shizuoka, Japan; 8 Department of Human Health Sciences, Graduate School of Medicine, Kyoto University, Kyoto, Japan; 9 Clinical Research Institute, National Hospital Organization Kyoto Medical Center, Kyoto, Japan; University of Frankfurt-University Hospital Frankfurt, Germany

## Abstract

The mechanisms that lead from obesity to atherosclerotic disease are not fully understood. Obesity involves angiogenesis in which vascular endothelial growth factor-A (VEGF-A) plays a key role. On the other hand, vascular endothelial growth factor-C (VEGF-C) plays a pivotal role in lymphangiogenesis. Circulating levels of VEGF-A and VEGF-C are elevated in sera from obese subjects. However, relationships of VEGF-C with atherosclerotic risk factors and atherosclerosis are unknown. We determined circulating levels of VEGF-A and VEGF-C in 423 consecutive subjects not receiving any drugs at the Health Evaluation Center. After adjusting for age and gender, VEGF-A levels were significantly and more strongly correlated with the body mass index (BMI) and waist circumference than VEGF-C. Conversely, VEGF-C levels were significantly and more closely correlated with metabolic (e.g., fasting plasma glucose, hemoglobin A1c, immunoreactive insulin, and the homeostasis model assessment of insulin resistance) and lipid parameters (e.g., triglycerides, total cholesterol (TC), low-density-lipoprotein cholesterol (LDL-C), and non-high-density-lipoprotein cholesterol (non-HDL-C)) than VEGF-A. Stepwise regression analyses revealed that independent determinants of VEGF-A were the BMI and age, whereas strong independent determinants of VEGF-C were age, triglycerides, and non-HDL-C. In apolipoprotein E-deficient mice fed a high-fat-diet (HFD) or normal chow (NC) for 16 weeks, levels of VEGF-A were not significantly different between the two groups. However, levels of VEGF-C were significantly higher in HFD mice with advanced atherosclerosis and marked hypercholesterolemia than NC mice. Furthermore, immunohistochemistry revealed that the expression of VEGF-C in atheromatous plaque of the aortic sinus was significantly intensified by feeding HFD compared to NC, while that of VEGF-A was not. In conclusion, these findings demonstrate that VEGF-C, rather than VEGF-A, is closely related to dyslipidemia and atherosclerosis.

## Introduction

Obesity plays a major role in the development of dyslipidemia, hypertension and many other sub-clinical abnormalities that contribute to the atherosclerotic process and onset of cardiovascular events [1], [2]. However, the mechanisms that lead from obesity to atherosclerosis and cardiovascular events are not fully understood.

It is widely accepted that adipose tissue development involves adipogenesis and angiogenesis [3]. Vascular endothelial growth factor-A (VEGF-A) signaling through VEGF receptor-2 (VEGFR-2) is the main angiogenic pathway [4]. It has been reported that VEGF-A accounts for much of the angiogenic activity of adipose tissue [5]. In addition, the administration of anti-VEGF-A antibody inhibited not only angiogenesis but also adipogenesis, which provides direct evidence that angiogenesis is essential for adipogenesis in obesity [6]. Circulating levels of VEGF-A are elevated in overweight and obese subjects [7]. Levels of VEGF-A is positively correlated with body mass index (BMI), and this correlation is apparently disconnected from insulin sensitivity [8]. However, a population-based cross-sectional study revealed that circulating VEGF-A levels have only a minor impact on the development of atherosclerosis [9].

Vascular endothelial growth factor-C (VEGF-C), a homologue of VEGF-A, plays a key role in lymphangiogenesis via VEGF receptor-3 (VEGFR-3). Deletion of *Vegfc* in mice leads to a complete absence of lymph vessels and embryonic lethality [10]. Overexpression of VEGF-C in the skin of transgenic mice induces selective hyperplasia of the lymphatic vasculature [11]. In the clinical setting, serum levels of VEGF-C are increased in patients with some cancers and are suggested to be associated with lymph node and distant metastases, as well as a poor prognosis [12]–[16]. Serum levels of VEGF-C are also elevated in overweight and obese subjects [7]. However, precise relationships of serum VEGF-C levels with clinical, lipid, and metabolic profiles and atherosclerosis are unknown.

Therefore, in the present study, we examined: 1) circulating levels of VEGF-A and VEGF-C in subjects not receiving any medications and examined their association with clinical, lipid, and metabolic parameters in comparison with those of VEGF-A, and 2) serum levels of VEGF-A and VEGF-C as well as their expression levels in the aortic sinus including atheromatous plaque in apolipoprotein E (apoE)-deficient mice fed a high-fat-diet in comparison with those fed normal chow.

## Methods

### Subjects

A cross-sectional study was carried out during a specified period from April 2008 to March 2011. A total of 423 Japanese subjects not receiving any medications were recruited in the Health Evaluation Center of Kyoto Medical Center. All participants provided written informed consent. The study protocol was approved by the Institutional Ethics Committee of Kyoto Medical Center.

### Data collection

Details are described elsewhere [17]. Briefly, blood was taken from the antecubital vein from 9 to 10 in the morning after a 12-h fast. Plasma levels of glucose and hemoglobin A1c (HbA1c), and serum levels of triglycerides, high-density-lipoprotein cholesterol (HDL-C), total cholesterol (TC), and low-density-lipoprotein cholesterol (LDL-C) were measured according to standard procedures. Non-high-density-lipoprotein cholesterol (nonHDL-C) was calculated employing the following formula: Non-HDL-C  =  TC−HDL-C. Immunoreactive insulin was measured using an enzyme immunoassay with a commercially available kit (Tosoh, Tokyo, Japan). The serum and plasma obtained were divided into aliquots and stored at −80°C until being assayed for VEGF-A and VEGF-C. Their serum (VEGF-C) or plasma (VEGF-A) concentrations were measured employing specific, commercially available, enzyme-linked immunosorbent assay (ELISA) kits according to the manufacturers' instructions (Quantikine, R&D Systems, Minneapolis, Minnesota, USA). The sensitivities of the assays for VEGF-C and VEGF-A were 4.6 and 5.0 pg/ml, respectively. Inter-/intra-assay coefficients of variation of ELISA for VEGF-C and VEGF-A were 7.2/3.5 and 7.0/4.5%, respectively. These assays were performed by an investigator blinded to the sources of the samples.

### Experimental atherosclerosis

ApoE-deficient mice (129Ola × C57BL/6 mixed background) were a generous gift from Edward M. Rubin (University of California at Berkeley, Berkeley, California, USA) [18]. They were mated with C57BL/6 mice to produce F1 hybrids. The F1 apo E+/− mice were then backcrossed with C57BL/6 mice for 10 generations. Mice homogeneous for the apoE-null allele on a C57BL/6 background were subsequently generated. Male mice were used in the subsequent experiments. They were kept in a temperature-controlled facility under a 12-h light–dark cycle with free access to food and water. After being weaned at 4 weeks of age, mice were fed a normal chow diet (NC, Oriental Yeast Co., Ltd., Tokyo, Japan) until 6 weeks of age, when they were divided into an NC group and a high-fat-diet (HFD) group containing 40% fat and 0.15% cholesterol (Oriental Yeast). The experimental protocols were approved by the Ethics Committee for Animal Experiments of Kyoto University.

### Serum samples from mice

At the age of 22 weeks, blood was drawn from the inferior vena cava of anesthetized mice and serum was separated by centrifugation at 4°C and stored at −80°C. Serum levels of total cholesterol, LDL-C, HDL-C, and triglycerides were measured using the standard methods (Nagahama Life Science Laboratory, Shiga, Japan). Those of VEGF-A and VEGF-C were measured employing specific ELISA kits according to the manufacturers' instructions (Quantikine, R&D Systems, Minneapolis, Minnesota, USA for VEGF-A, Cusabio Biotech Co., Ltd., Newark, Delaware, USA for VEGF-C).

### Preparation of tissue and quantification of atherosclerosis in mice

After anesthesia, the mice were euthanized at 22 weeks of age, and their proximal aortas were excised, fixed in 4% paraformaldehyde (Nacalai Tesque, Inc, Kyoto, Japan), washed in sucrose, embedded in OCT compound (Tissue-Tek, Sakura Finetechnical Co., Ltd., Tokyo, Japan), frozen on dry ice, and then stored at −80°C until sectioning. The OCT-embedded aortas were sectioned with a cryostat, and 6-µm sections were obtained sequentially, beginning at the aortic valve. Eight sections obtained every 24 µm from the aortic sinus were stained with oil red O and used for quantification of the lesion areas. The total and atherosclerotic areas of each aorta were measured with image analysis (ImageJ), and the ratio of the atherosclerotic area to the total area was calculated.

### Immunohistochemistry

The frozen sections were washed in phosphate-buffered saline (PBS) and endogenous peroxidase activity was blocked by 0.3% H_2_O_2_ in methyl alcohol for 30 min. The sections were washed in PBS (6 times, 5 each min) and mounted with 1% normal goat serum in PBS for 30 min. Subsequently, primary antibody (rat anti-mouse VEGF-A antibody (1∶100), Biolegend, San Diego California, USA; rabbit anti-rat VEGF-C antibody (1∶200) (also reacts with mouse and human VEGF-C), Abcam plc., Tokyo, Japan) was applied overnight at 4°C. After washing in PBS (6 times, 5 min), they were incubated with peroxidase-labeled secondary antibody polymer (Histofine Simple Stain Mouse MAX-PO (Rat or Rabbit), Nichirei Biosciences Inc., Tokyo, Japan) for 30 min. After washing in PBS (6 times, 5 min), a coloring reaction was carried out with diaminobenzidine (Wako Pure Chemical Industries, Osaka, Japan) and nuclei were counterstained with hematoxylin. The numbers of VEGF-A-positive and VEGF-C-positive cells were counted in a cross-section of the aortic sinus including atheromatous plaque in each mouse.

### Statistical analysis

All statistical analyses were performed using Stat View version 5.0 for Windows (SAS Institute Inc., Cary, North Carolina, USA.). The Mann-Whitney U test was employed for comparisons of values between the two groups. Relationships between either of VEGF-A or VEGF-C and other parameters were analyzed by age- and gender-adjusted correlations and a stepwise linear regression. Stepwise regression was performed in a forward direction with *F* for the entry set to 4. Because triglycerides, fasting glucose, immunoreactive insulin, homeostasis model assessment of insulin resistance (HOMA-IR), adiponectin, high-sensitivity C-reactive protein (hsCRP), and VEGF-A were normally distributed after logarithmic transformation, the logarithms of these parameters were used in the analyses. Data are expressed as the means ± SD or the medians and inter-quartile ranges, as appropriate. Values of *P*<0.05 were considered significant.

## Results

### Differential Association of Circulating Levels of VEGF-C and VEGF-A with Clinical, Lipid, and Metabolic Parameters

The clinical characteristics of subjects are shown in Table 1. The prevalence of obesity (defined as a body mass index >25 kg/m^2^), hypertension (defined as systolic blood pressure > = 140 or diastolic blood pressure > = 90 mmHg), dyslipidemia (defined as LDL-C > = 140 mg/dL, HDL-C<40 mg/dL, or triglycerides > = 150 mg/dL) was 26, 13, and 42%, respectively. That of metabolic syndrome (defined as the presence of any 3 of the following 5 criteria: 1) increased waist circumference (> = 85 cm in men or > = 90 cm in women), 2) elevated triglycerides (> = 150 mg/dL), 3) reduced concentration of high-density lipoprotein cholesterol (HDL-C) (<40 mg/dL in men or <50 mg/dL in women), 4) elevated blood pressure (systolic blood pressure > = 130 or diastolic blood pressure > = 85 mmHg), and 5) elevated fasting glucose (> = 100 mg/dL)) was 16%. Thus, they were not necessarily healthy, but had yet to receive any medications. Distribution of circulating levels of VEGF-A were skewed, while levels of VEGF-C were almost normally distributed. Therefore, values of VEGF-A levels were log-transformed for subsequent analyses.

**Table 1:** Demographic Data of Human Subjects.

Number of patients, n	423
(Male/Female, n)	(281/142)
Age, y	45±9
Male gender,%	66±47
Body mass index, kg/m^2^	22.9±3.1
Waist circumference, cm	83±9
Systolic blood pressure, mmHg	117±16
Diastolic blood pressure, mmHg	74±11
Fasting plasma glucose, mg/dL	95 [90–101]
Hemoglobin A1c,%	5.19±0.29
Immunoreactive insulin, mU/L	5.0 [4.0–8.0]
HOMA-IR	1.3 [0.8–1.8]
Triglycerides, mg/dL	96 [66–138]
HDL-C, mg/dL	69±18
Total cholesterol, mg/dL	209±32
LDL-C, mg/dL	126±31
Non-HDL-C, mg/dL	139±36
hsCRP, µg/mL	0.15 [0.10–0.30]
Adiponectin, µg/mL	7.7 [5.7–10.9]
VEGF-A, pg/mL	278 [163–434]
VEGF-C, pg/mL	6135±1409

Data are expressed as the mean ± SD, median [25–75 percentile], or number of patients. HDL-C: high-density lipoprotein cholesterol; LDL-C: low-density lipoprotein cholesterol; Non-HDL-C: non-high-density lipoprotein cholesterol; HOMA-IR: homeostasis model assessment of insulin resistance; hsCRP: high-sensitivity C-reactive protein; VEGF-A: vascular endothelial growth factor-A, VEGF-C: vascular endothelial growth factor-C.

Then, we examined the association of circulating VEGF-A and VEGF-C levels with clinical, lipid, and metabolic parameters after adjusting for age and gender (Table 2). Levels of VEGF-A were significantly and more strongly correlated with the body mass index, waist circumference, and adiponectin levels than VEGF-C. Conversely, levels of VEGF-C were significantly and more closely correlated with lipid (e.g., triglyceride, TC, LDL-C, and non-HDL-C) and metabolic parameters (e.g., fasting plasma glucose, hemoglobin A1c, immunoreactive insulin, and HOMA-IR).

**Table 2:** Correlations of Vascular Endothelial Growth Factor-A (VEGF-A) and Vascular Endothelial Growth Factor–C (VEGF-C) with Other Parameters.

	VEGF-A		VEGF-C	
	*r*	*P*	*r*	*P*
Body mass index , kg/m^2^	0.21	<0.0001	0.13	0.008
Waist circumference, cm	0.19	0.0001	0.13	0.007
Systolic blood pressure , mmHg	0.09	0.06	0.08	0.09
Diastolic blood pressure , mmHg	0.16	0.001	0.08	0.09
Fasting plasma glucose, mg/dL a	0.09	0.06	0.11	0.03
Hemoglobin A1c,%	0.11	0.02	0.13	0.007
Immunoreactive insulin, mU/L a	0.08	0.1	0.17	0.0006
HOMA-IR a	0.09	0.07	0.18	0.0003
Triglycerides, mg/dL a	0.10	0.04	0.23	<0.0001
HDL-C, mg/dL	−0.09	0.054	−0.08	0.1
Total cholesterol, mg/dL	0.07	0.2	0.18	0.0002
LDL-C, mg/dL	0.07	0.1	0.17	0.0004
Non-HDL-C, mg/dL	0.11	0.03	0.20	<0.0001
hsCRP, ng/mL a	0.10	0.04	0.12	0.01
Adiponectin, µg/mL a	−0.14	0.003	−0.08	0.1
VEGF-A, pg/mL a	-	-	0.11	0.03
VEGF-C, pg/mL	0.11	0.03	-	-

Abbreviations used in this table are the same as in Table 1.

a Log-transformed to obtain normal distributions. Values were adjusted for age and gender.

### Independent Determinants of VEGF-A and VEGF-C levels

**Figure 1:**
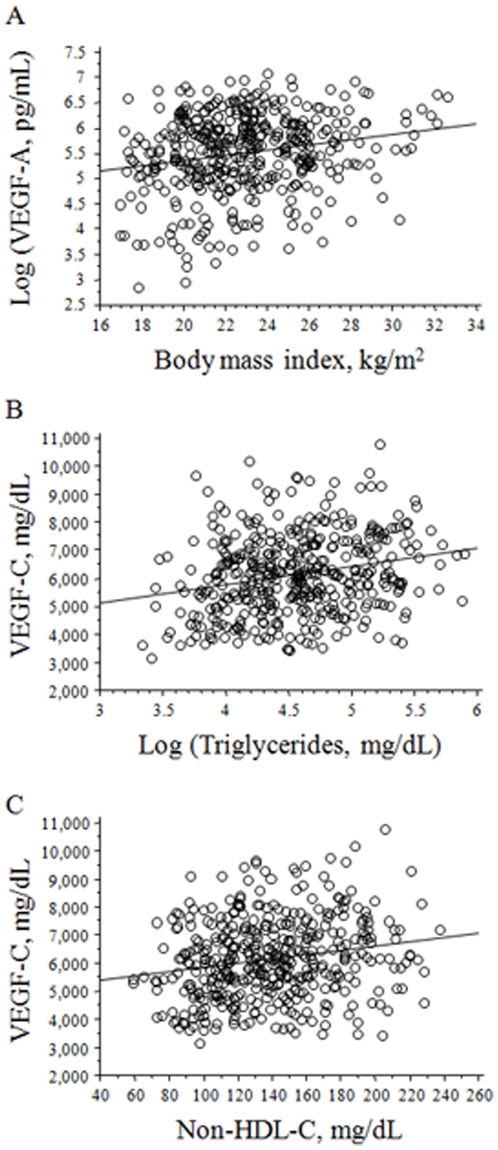
The correlation of circulating vascular endothelial growth factor-A (VEGF-A) or C (VEGF-C) levels with their independent determinants. A. The correlation between circulating VEGF-A levels and the body mass index. B. The correlation between those of VEGF-C and those of triglycerides. C. The correlation between those of VEGF-C and those of non-high-density-lipoprotein cholesterol (nonHDL-C).

To identify independent determinants of VEGF-A and VEGF-C levels, stepwise multiple regression analyses were performed. The body mass index and age were independent determinants of VEGF-A levels (Table 3). In contrast, independent determinants of VEGF-C were age, triglycerides, non-HDL-C, and hemoglobin A1c (Table 3). These findings suggest that VEGF-A is associated with overweightness itself, whereas VEGF-C is closely associated with lipid and metabolic disorders. The correlation between VEGF-A and the body mass index and correlations of VEGF-C with triglycerides and non-HDL-C are shown in Figures 1A, B, and C, respectively.

**Table 3:** Independent determinants of VEGF-A and VEGF-C levels.

	VEGF-A			VEGF-C		
	*β*	SEM	*F*	*β*	SEM	*F*
Body mass index, kg/m^2^	0.16	3.4	11			
Age, y	0.11	0.1	5	−0.18	8.1	13.1
Triglycerides, mg/dL^ a^				0.14	1.3	6.2
Non-HDL-C, mg/dL				0.14	2.2	5.7
Hemoglobin A1c, %				0.11	251	4.3

Abbreviations used in this table are the same as in Table 1. These models include data on the age, a male gender, body mass index, waist circumference, systolic and diastolic blood pressures, fasting plasma glucose, hemoglobin A1c, immunoreactive insulin, HOMA-IR, triglycerides, HDL-C, total cholesterol, LDL-C, non-HDL-C, hsCRP, and adiponectin.

### Serum and expression levels in atheromatous plaque of VEGF-A and VEGF-C in apoE-deficient mice

**Figure 2:**
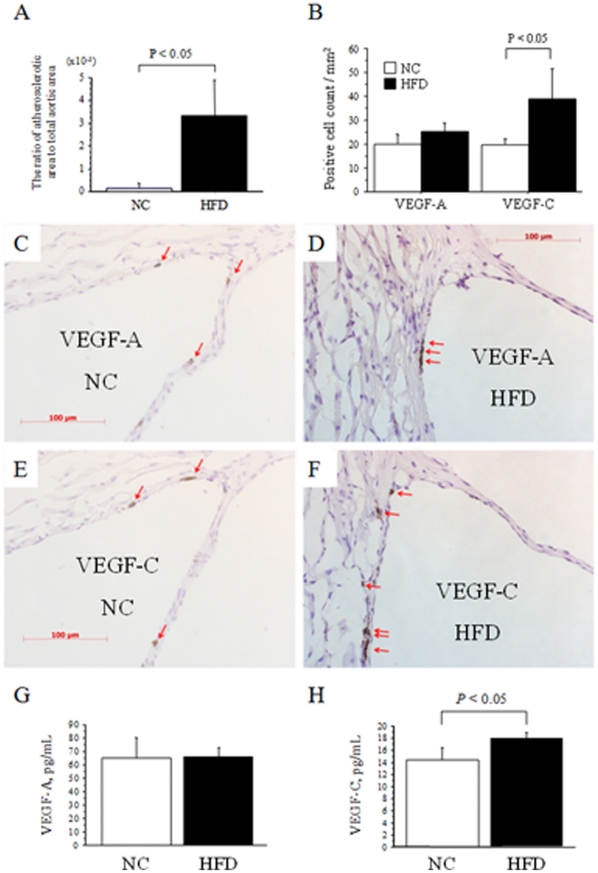
Serum and expression levels in atheromatous plaque of VEGF-A and VEGF-C in apoE-deficient mice. A. Quantification of the lesion size in the proximal aortas of apolipoprotein E (apoE)-deficient mice fed normal chow (NC, n = 3) or a high-fat-diet (HFD, n = 3). The ratio of the atherosclerotic area to the total area was significantly greater in HFD than NC mice. B. Quantification of the expression of vascular endothelial growth factor-A (VEGF-A) and vascular endothelial growth factor-C (VEGF-C) in NC and HFD mice. The expression of VEGF-C, but not VEGF-A, was significantly intensified by feeding HFD compared to NC. C–F. Representative microscopic views (x400) of the expression of VEGF-A in the aortic sinus of apoE-deficient mice fed NC (C) or a HFD (D), and those of VEGF-C in NC (E) or HFD (F) mice. The red arrows indicate VEGF-A- or VEGF-C-positive cells. G and H. Serum levels of VEGF-A (G) and VEGF-C (H) in apoE-deficient mice fed a HFD or NC for 16 weeks. The data are means ± SD.

To examine the relationship between VEGF-C and atherosclerosis with dyslipidemia, apoE-deficient mice, one of the most popular animal models of dyslipidemia and atherosclerosis, were fed a HFD (n = 3) or NC (n = 3) for 16 weeks. Thereafter, body weight (42±1 vs. 31±1 g, respectively, *P*<0.05) and serum levels of total cholesterol (1137±237 vs. 685±125 mg/dL, respectively, *P*<0.05) and LDL-C (393±78 vs. 162±21 mg/dL, respectively, *P*<0.05), but not HDL-C (28±5 vs. 20±3 mg/dL, respectively) and triglycerides (122±37 vs. 127±60 mg/dL, respectively), were significantly higher in the HFD than NC group. Atheromatous plaque in proximal aortas quantified by oil red O staining was markedly greater in the HFD than NC group (Figure 2A). Immunohistochemistry revealed that the number of VEGF-C-positive cells, but not that of VEGF-A, was significantly greater in HFD with advanced atherosclerosis than NC mice with minimal atherosclerosis (Figures 2B–F). Interestingly, serum levels of VEGF-C, but not those of VEGF-A, were significantly higher in HFD than NC mice (Figures 2G and H). These findings indicate that VEGF-C, rather than VEGF-A, is closely related to advanced atherosclerosis with marked hypercholesterolemia induced by HFD in apoE-deficient mice.

## Discussion

The present study demonstrated that circulating levels of VEGF-C are closely associated with dyslipidemia in marked contrast to the fact that the strongest independent determinants of VEGF-A was the body mass index. These findings suggest that VEGF-A increases in association with overweightness itself; however, VEGF-C increases in association with dyslipidemia rather than overweightness per se. To our knowledge, this is the first study to report an association between VEGF-C and dyslipidemia.

Loebig et al. demonstrated that a positive correlation between VEGF-A and body mass index and that the relationship is apparently disconnected from insulin sensitivity [8]. Sandhofer et al. have shown that circulating VEGF-A levels have only a minor impact on the development of atherosclerosis [9]. However, VEGF-C is tightly associated with dyslipidemia, a potent risk factor as well as a therapeutic target of cardiovascular disease. In addition, we demonstrated that serum levels and expression levels in atheromatous plaque of VEGF-C, but not VEGF-A, were significantly increased in HFD-fed apoE-deficient mice with advanced atherosclerosis, suggesting that VEGF-C was more closely related to atherosclerosis with dyslipidemia than VEGF-A. Therefore, VEGF-C might have more impact on atherosclerosis and future cardiovascular events than VEGF-A in humans.

VEGF-C induces lymphangiogenesis, which is involved in the draining of interstitial fluid and in immune function and inflammation [11]. It has been reported that VEGF-C levels are elevated in patients with refractory hypertension, and that VEGF-C/VEGFR-3 signaling in macrophages is a major determinant of the extracellular volume and blood pressure homeostasis [19]. The trapping of VEGF-C by soluble VEGF receptor-3 blocks VEGF-C signaling, and elevates the blood pressure in response to a high-salt-diet [19]. Thus, VEGF-C seems to be up-regulated to compensate for salt-diet-induced hypertension. Similarly, VEGF-C might be up-regulated to compensate for the development and progression of atheromatous plaque by draining lipid and/or inflammatory cells in response to dyslipidemia. However, further investigation is required regarding this matter.

While lymphatic vessels are rare in the atherosclerotic intima [20], membrane-bound VEGF receptor-2 (VEGFR-2) is up-regulated in atherosclerotic lesion in human coronary arteries [21]. A recent report suggested that a soluble form of VEGFR-2 (sVEGFR-2) inhibits lymphangiogenesis by blocking the VEGF-C function, and that the tissue-specific loss of the sVEGFR-2 gene induces lymphatic invasion of the normally alymphatic cornea and hyperplasia of skin lymphatics without affecting the blood vasculature [22]. These findings suggest that naturally occurring sVEGFR-2 acts as a molecular uncoupler of blood and lymphatic vessels [22]. We recently demonstrated that serum levels of sVEGFR-2 are increased in sera from subjects with metabolic syndrome in association with insulin resistance [17]. Thus, it is of interest to elucidate the interaction between VEGF-C and sVEGFR-2 in the regulation of lymphangiogenesis at vessel walls and in the progression of atherosclerosis.

Several study limitations should be considered. First, the present study was limited due to its moderate sample size. However, all the study participants did not receive any medications including statins or renin-angiotensin-system inhibitors, which could substantially affect serum levels of angiogenesis-related factors. Thus, the relationships between these biomarkers and established risk factors in this study are physiological. Second, this human study was cross-sectional, and, thus, the results cannot help answer the question of whether elevations of VEGF-C are merely consequences of metabolic abnormality or causes of future cardiovascular events in these subjects. Third, to elucidate its prognostic significance, the direct relationship of VEGF-C with cardiovascular events and/or atherosclerosis in patients should be investigated in future studies. Finally, at present, the sources of endogenous VEGF-C in human sera, and the relationships of their levels with cardiovascular lymphangiogenic activity are unclear.

Nevertheless, the present study first demonstrates that circulating levels of VEGF-C are closely associated with dyslipidemia and atherosclerosis. Future investigations are warranted to determine the precise role of lymphangiogenesis in the pathogenesis of atherosclerosis, and the clinical utility of serum VEGF-C levels.
